# Clonal and resistance profiles of fluoroquinolone-resistant uropathogenic *Escherichia coli* in countries with different practices of antibiotic prescription

**DOI:** 10.3389/fmicb.2024.1446818

**Published:** 2024-10-02

**Authors:** Debarati Choudhury, Rawan Alanbari, Pauline Saveliev, Evgeni Sokurenko, Miklos Fuzi, Veronika Tchesnokova

**Affiliations:** ^1^Department of Microbiology, University of Washington School of Medicine, Seattle, WA, United States; ^2^Department of Microbiology, Al-Mustansiriyah University, College of Medicine, Baghdad, Iraq; ^3^Khoury College of Computer Sciences, Northeastern University, Boston, MA, United States; ^4^Independent Researcher, Seattle, WA, United States

**Keywords:** uropathogenic *Escherichia coli*, fluoroquinolone, antibiotic resistance, clonality, antibiotic use

## Abstract

**Background:**

Antibiotic prescription practices differ between countries, influencing regional antimicrobial resistance prevalence. However, comparisons of clonal diversity among resistant bacteria in countries with different prescribing practices are rare. The rise of fluoroquinolone-resistant *Escherichia coli* (FQREC), often multidrug-resistant, exacerbates global antibiotic resistance. Unlike in the USA, antibiotics are commonly dispensed in Iraq without prescriptions, leading to widespread overuse and misuse. This study aimed to assess the impact of varying antibiotic use practices on FQREC diversity.

**Methods:**

We compared FQREC prevalence, multidrug resistance, and clonality of FQREC among *E. coli* isolated from urine submitted between 2017 and 2018 to three US hospitals and two Iraqi hospitals. All FQREC isolates were analyzed for QRDR mutations and the presence of PMQR genes. A subset of FQREC strains from the ST131-*H*30R/Rx subgroups underwent whole-genome sequencing (WGS) and phylogenetic analysis.

**Results:**

*E. coli* from Iraq showed significantly higher resistance to all tested antibiotics compared to those from the USA, with 76.2% being FQREC versus 31.2% in the USA (*p* < 0.01). Iraqi FQREC strains were more frequently multidrug resistant. The predominant subgroup in both countries was ST131-*H*30, with the notable absence of ST1193 among Iraqi FQREC. Iraqi-origin ST131-*H*30 strains exhibited higher minimum inhibitory concentrations (MICs) for ciprofloxacin and greater resistance to third-generation cephalosporins (3GC), trimethoprim/sulfamethoxazole (TMP/STX), and imipenem (IMI) than those from the USA. Increased 3GC resistance in Iraqi strains was linked to a higher proportion of *bla*_CTX-M-15_-carrying *H*30Rx subclade isolates. Additionally, Iraqi *H*30 strains exhibited higher MICs for fluoroquinolones due to more frequent carriage of PMQR determinants compared to US strains. Whole-genome sequencing was performed on 46 Iraqi and 63 US *H*30 isolates. Phylogenetic analysis revealed two clades—*H*30R and *H*30Rx—present in both countries, with isolates from both regions distributed throughout, without the emergence of distinct new major subclones. However, Iraqi isolates tended to cluster in separate subclades, indicating endemic circulation of the strain groups.

**Conclusion:**

In regions like Iraq, where antibiotics are overused and misused, resistance among uropathogenic *E. coli* to various antibiotics is significantly higher. Most Iraqi resistant strains belong to well-known international groups, and no new highly successful strains have emerged. The absence of ST1193 in Iraq may reflect regional, socioeconomic, demographic, or cultural factors that hinder the success of certain strain groups in the country.

## 1 Introduction

Antibiotic resistance (AMR) in pathogenic bacteria is one of our biggest public health challenges, with serious political and economic consequences (Connor et al., 2023). Annually, resistant bacteria cause more than 700,000 deaths globally, and this number is predicted to reach 10 million by 2050 (Tang et al., 2023). Antibiotic prescription practices—and thus the extent of their overuse and misuse—vary vastly between countries, resulting in significant regional differences in the prevalence of antimicrobial resistance (Mortazavi-Tabatabaei et al., 2019). Generally, antibiotic-resistant bacteria are considered to be clonal, i.e., they form and circulate in closely related strain groups (Petty et al., 2014). However, it remains unclear how different antibiotic prescription practices affect the clonal diversity of the resistant bacteria. One way to understand that is to compare the clonal diversity of resistant bacteria in countries with different antibiotic usage patterns at the same time, but such head-to-head studies have not been performed.

Urinary tract infections (UTIs) are the most common bacterial infections managed in general medical practice, almost universally treated with antibiotics. *Escherichia coli* is the main cause of UTI and, thus, is among the most common human pathogens treated with antibiotics (Tchesnokova et al., 2019a). Ciprofloxacin (CIP) and other fluoroquinolones (FQs) are among the most frequently prescribed antibiotics for UTI treatment (Conley et al., 2018; Waller et al., 2018).

The rise of fluoroquinolone-resistant *E. coli* (FQREC), which are often multidrug-resistant, is a major contributor to the global antibiotic resistance pandemic. The FQREC occurrence is strongly associated with hospitalization and mortality rates from sepsis (Spellberg and Doi, 2015). The FQREC spread is dominated by a limited number of highly successful international clonal lineages (Price et al., 2013). Strains from two multidrug-resistant clonal groups (sequence types, STs) are dominant among UTI-associated FQREC – those belonging to subclades *H*30R and *H*30Rx (aka C1 and C2, respectively) of ST131 (*E. coli H*30) that emerged in the late 1990s and to a more recently emerged ST1193 (Johnson et al., 2013; Price et al., 2013; Johnson et al., 2018; Tchesnokova et al., 2019b). Currently, both *E. coli H*30 and ST1193 are pandemic and account for 60–80% of CIP-R *E. coli* found in US patients with UTIs (Tchesnokova et al., 2020). Several international but smaller, high-risk clonal groups, such as ST405, ST410, ST648, ST10, and ST69, also contribute to antibiotic resistance (Roy Chowdhury et al., 2018; Connor et al., 2023).

CIP resistance is primarily associated with point mutations in the quinolone resistance-determining regions (QRDR) of chromosomal genes gyrA and parC, which encode the main targets of FQ-bacterial DNA topoisomerases: GyrA (residues S83 and D87) and ParC (residues S80 and E84) (Hooper and Jacoby, 2015). A ‘classical’ set of at least three QRDR mutations – of amino acids S83 and S87 in GyrA and S80 in ParC – results in the clinically relevant resistance level of at least two mg/L (Fuzi et al., 2017). The CIP-R phenotype is also mediated by genes on mobile elements, so-called plasmid-mediated quinolone resistance (PMQR) genes (Jacoby et al., 2014; Ruiz, 2019).

In this study, we compared the prevalence, multidrug resistance, and clonality of FQREC strains among *E. coli* isolated from urine submitted in 2017–2018 to clinical microbiology laboratories in Bagdad, Iraq, and three cities across the USA – Seattle, Los Angeles, and New York. Unlike in the USA, antibiotics in Iraq are commonly dispersed by pharmacies without a doctor’s prescription, leading to widespread overuse of broad-spectrum antibiotics (Alkadhimi et al., 2020; Kurmanji et al., 2021).

## 2 Methods

### 2.1 Collection of clinical urine samples with *Escherichia coli*

Urinary *E. coli* were collected from two hospitals in Iraq (Al-Emammian Al-Kadhimi Teaching Hospital and Al-Kindy Teaching Hospital, Baghdad) and from three hospitals in the US (Harborview Medical Center, Seattle, WA; Los Angeles County and University of South California Medical Center, CA; New York University Langone Medical Center, NY) from January 2017 to February 2018. The Iraqi *E. coli* were isolated as follows: 143 urine samples from both hospitals were processed in the same clinical laboratory according to standard practice. Plates with potential *E. coli* growth were identified, and colonies showing mixed characteristics were collected into an Amies Gel transportation vial (BD BBL^™^ CultureSwab^™^ Plus) and sent to the University of Washington (UW) (Seattle, WA, United States). The US *E. coli* were isolated as part of the multicenter surveillance study carried out by ID Genomics and UW, as described by Tchesnokova et al. (2018). 940 US urine samples (Harborview Medical Center, Seattle, *n* = 519; Los Angeles County and University of South California Medical Center, CA, *n* = 224 and New York University Langone Medical Center, NY, *n* = 197) were processed in clinical labs in each hospital according to their standard practices, and 5–10 individual colonies were collected into the same transportation tubes and sent to ID Genomics and UW. *E. coli* from all urine samples (from both Iraq and the USA) were obtained in a de-identified manner. In both cases, the clinical samples were provided randomly within consecutive days of submission and were sourced from patients with suspected UTIs, with clinical and laboratory diagnosis conducted according to similar standards in both countries.

### 2.2 Isolation and susceptibility testing of *Escherichia coli* from clinical urine

Each sample was streaked onto a UTI agar plate (HardyCHROM^™^ UTI, Chromogenic Medium) to morphologically confirm the presence of *E. coli* as the sole organism in the sample. When the presence of different species was suspected, 4–5 morphologically distinct *E. coli*-like colonies were re-isolated for further analysis. Saved *E. coli* were tested for resistance to a set of antibiotics using the standard disk diffusion method according to CLSI standard procedure (Clinical and Laboratory Standards Institute, 2022). If the test revealed the presence of a heterogeneous population, the sample was subcultured to isolate distinct colonies. The antibiotics used were ciprofloxacin (CIP), ceftazidime (a third-generation cephalosporin, 3GC), trimethoprim/sulfamethoxazole (TMP/SXT), nitrofurantoin (NIT), imipenem (IMI), and fosfomycin (FOS) (Hardy Diagnostic, United States). Minimum inhibitory concentration (MIC) was determined for ciprofloxacin, ceftazidime, and ceftriaxone (Thermo Fisher Scientific, United States) using agar dilution method as described in Clinical and Laboratory Standards Institute (2022) for all ST131-*H*30 *E. coli* isolates. The ciprofloxacin concentration used to measure high MIC values ranged from 0.25 mg/L to 2048 mg/L. In case the isolates continued to grow beyond the highest tested concentration of antibiotics, they were assigned the next value.

### 2.3 *Escherichia coli* clonal typing

All *E. coli* were subjected to 7SNP-based clonal typing, as described by Tchesnokova et al. (2016). Additionally, all *E. coli* were tested using *H*30-*specific* probes described, also described by Tchesnokova et al. (2016). To assign sequence types, four genes (*fumC, fimH, gyrA, parC*) were sequenced in all Iraqi *E. coli* isolates and a randomly selected subset of US isolates, following the methodology of Weissman et al. (2012). The primers and probes used in this study are detailed in Supplementary Table S4.

### 2.4 Whole genome sequencing

All ST131-*H*30 isolates were subjected to whole genome sequencing on the Illumina MiSeq platform using the MiSeq 600 cycles v3 kit (Illumina, United States). Raw sequencing data were uploaded to the Enterobase database^1^ for genome assembly (Supplementary Data S5) and allele assignment. Core gene alleles were downloaded and analyzed for sequence identity. Phylogenetic trees were built using MEGA 7 software. For isolates placed at the same branch, wgMLST was downloaded and analyzed for differences in sequence content (Supplementary Data S4).

The maximum-likelihood tree was constructed using 1,043 core genes (see Supplementary Data S1; Supplementary Data S2), following a methodology that considered any allelic change in the core genome as a single difference between isolates instead of counting the total number of SNPs. Since all isolates were resistant to CIP and carried a full set of four QRDR mutations (GyrA S83L-D87N and ParC S80I-E84G), a CIP-sensitive ST131-*H*30 strain without QRDR mutations was used to root the tree (Enterobase Uberstrain ID ESC_CB6620AA). The *H*30R/*R*x split was confirmed by the presence of 260–264 SNPs (Price et al., 2013).

### 2.5 Identification of resistance determinants

Beta-lactamase determinants, QRDR mutations, and PMQR loci were identified on assembled genomes using the Bacterial and Viral Bioinformatics Resource Center^2^ and NCBI Blast Tool.^3^

### 2.6 Statistical analysis

Differences in the prevalence of non-susceptible *E. coli* and individual clonal groups were estimated using 2×2 comparisons with the chi-square test or logistic regression, as indicated. MIC data were analyzed as log_2_[MIC] values in linear regression, with averages and standard errors reported. All analyses were conducted using Stata v14 software (StataCorp, TX, United States).

## 3 Results

### 3.1 Resistance of urine-derived *Escherichia coli* to commonly used antibiotics

Overall, we analyzed the antibiotic resistance of urine *E. coli* isolated from patients with suspected UTI from sequential samples submitted for analysis to clinical microbiology laboratories in two Bagdad (Iraq) hospitals (143 isolates) and three USA hospitals in Seattle, Los Angeles, and New York City (940 isolates), all between 2017 and 2018 (see Supplementary Data S3). Approximately three-quarters of urine *E. coli* from Iraq displayed resistance to ciprofloxacin (CIP), trimethoprim/sulfamethoxazole (TMP/SXT), and third-generation cephalosporins (3GC), with resistance rates 2.5, 2.1, and 5.1 times higher, respectively, than those observed in US samples (*p* < 0.001) (Table 1). Furthermore, resistance to nitrofurantoin (NIT) and fosfomycin (FOS) was 4.4 and 2.4 times higher, respectively, in Iraq samples compared to US samples (*p* < 0.001). Additionally, there were eight samples from Iraq (5.6%) carrying *E. coli* resistant to imipenem (IMI), whereas none were found in US samples (*p* < 0.001). Among Iraq-derived *E. coli*, 87% of isolates were resistant to three or more antibiotic classes, i.e., they should be defined as multidrug-resistant (MDR) isolates (Table 1). Among the US-derived isolates, only 38.0% of isolates (*p* < 0.001) could be classified as MDR. Moreover, 72.7 and 49.7% of isolates from Iraq were resistant to four and five antibiotics, respectively, compared to only 20.4 and 7.9% of isolates from the US (*p* < 0.001 both). No isolates from either country proved resistant to all of the tested antibiotics.

**Table 1:** Antibiotic resistance of urinary *E. coli* from Iraq hospitals has increased significantly compared to *E. coli* from US hospitals, overall, for ciprofloxacin-sensitive (CIP-S) and resistant (CIP-R) subpopulations, and for *H*30 and non-*H*30 CIP-R clones.

*E. coli* collection		All samples			CIP-S *E. coli*-carrying samples			CIP-R *E. coli*-carrying samples			CIP-R vs. CIP-S, *p* value^b^	
		Iraq	USA	*p* value^a^	Iraq	USA	*p* value^a^	Iraq	USA	*p* value^a^	Iraq	USA
No. (% All)		143	940		34 (23.8)	647 (68.8)	**<0.0001**	109 (76.2)	293 (31.2)	**<0.0001**	**<0.0001**	**<0.0001**
Resistance, %	3GC^c^	76	15	**<0.0001**	41.2	4.9	**<0.0001**	86.2	37.9	**<0.0001**	**<0.0001**	**<0.0001**
	TMP/STX^c^	75	36	**<0.0001**	58.8	27.4	**0.0001**	79.8	56.3	**<0.0001**	**0.0138**	**<0.0001**
	CIP^c^	76	31	**<0.0001**	0	0	n**/a**	100	100	n/a	n/a	n/a
	NIT^c^	14	3.2	**<0.0001**	8.8	1.5	**0.0025**	15.6	6.8	**0.0068**	0.3199	**<0.0001**
	IMI^c^	5.6	0	**<0.0001**	0	0	n/a	7.3	0	**<0.0001**	0.1052	n/a
	FOS^c^	9.1	3.8	**0.0048**	5.9	3.7	0.5193	10.1	4.1	**0.0214**	0.4562	0.7751
	MDR-3^c^	87	38	**<0.0001**	86	39	**<0.0001**	95	28	**<0.0001**	0.2701	0.0362
	MDR-4^c^	73	20	**<0.0001**	78	24	**<0.0001**	40	0	**<0.0001**	**0.0004**	**<0.0001**
	MDR-5^c^	50	8	**<0.0001**	65	9	**<0.0001**	19	4	**<0.0001**	**<0.0001**	**0.0109**

a Prevalence and resistance compared between Iraq and US urine samples using the chi-square test (*p* < 0.05 in bold).

b Prevalence and resistance were compared between CIP-S vs. CIP-R *E. coli* within either Iraqi or US samples using the chi-square test (*p* < 0.05 in bold).

c 3GC, 3rd generation cephalosporins, TMP/STX, trimethoprim/sulfamethoxazole, CIP, ciprofloxacin, NIT, nitrofurantoin, IMI, imipenem, FOS, fosfomycin; MDR-3, MDR-4 and MDR-5—multidrug-resistant to at least 3, 4 and 5 classes of antibiotics (including ampicillin/amoxiclav and tetracycline). The sample is considered resistant if at least one of the isolated *E. coli* is non-susceptible to an antibiotic.

Consistent with the overall resistance pattern, within both the CIP-resistant (CIP-R) and CIP-sensitive (CIP-S) subpopulations, resistance rates in Iraq were significantly higher than in the US for all antibiotics except FOS (*p* < 0.01). At the same time, in both Iraqi and US collections, the CIP-R *E. coli* exhibited significantly higher resistance to 3GC and TMP/SXT compared to the CIP-S *E. coli* (see Table 1; *p* < 0.05). Among the US-derived *E. coli*, CIP-R *E. coli* were also more resistant to NIT compared to CIP-S samples (*p* < 0.001). There were significantly more MDR isolates among CIP-R than CIP-S isolates, even without considering the resistance to CIP.

### 3.2 Clonal and QRDR mutations diversity of CIP-R *Escherichia coli*

**Figure 1:**
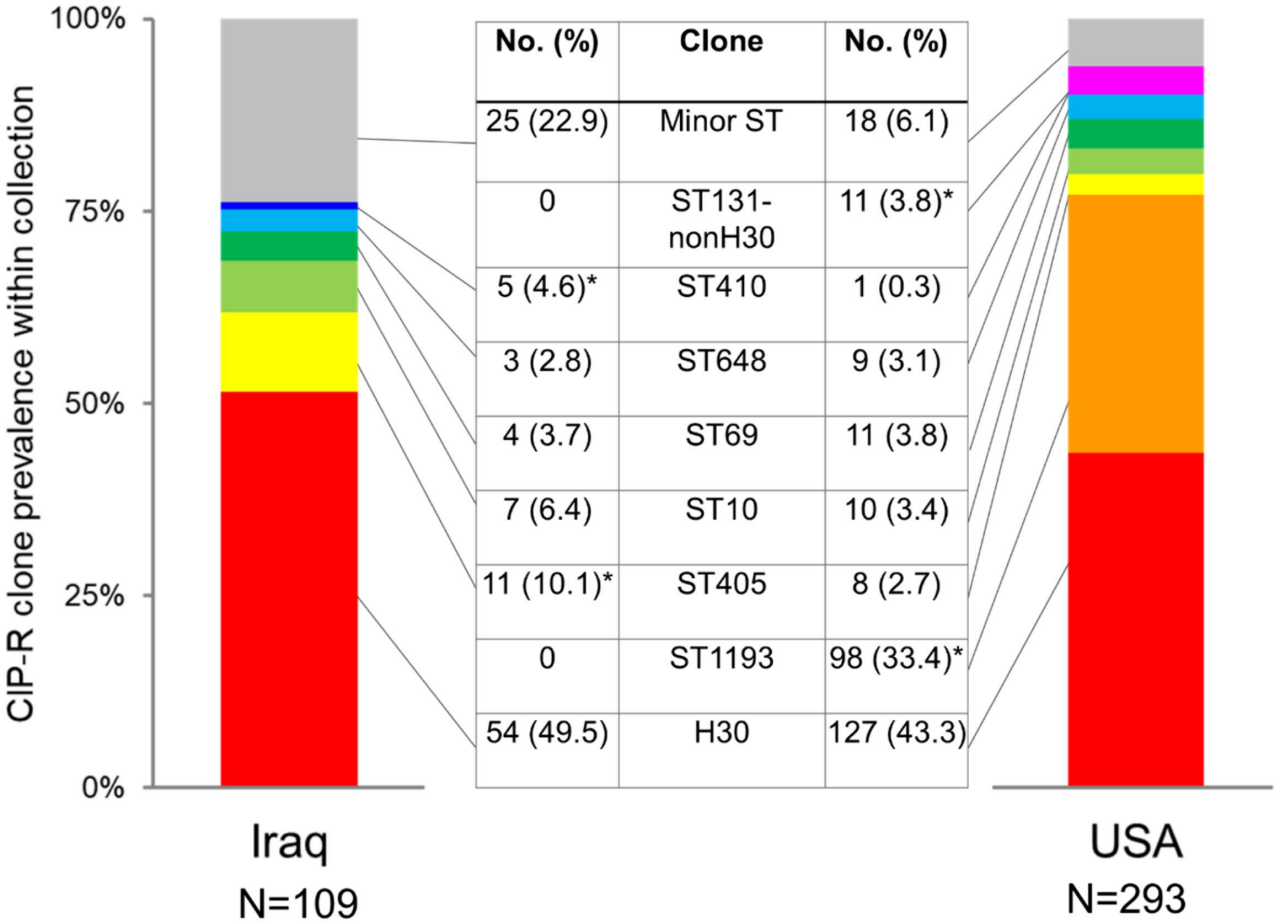
Comparison of prevalence of major CIP-R *E. coli* clones isolated from Iraq and US urine samples. Star (*) indicates a statistically higher prevalence of a clone (*p* < 0.05 in the chi-square test).

The four-gene clonotyping scheme identified 22 clonal groups among 109 CIP-R isolates from Iraq and 23 groups among 293 CIP-R isolates from the US (Supplementary Table S1). The group size is highly diverse in both countries, with 16 and 15 clonal groups in Iraq and the USA, respectively, being relatively minor, comprising <3% of CIP-R isolates each (Figure 1; Supplementary Table S1). Still, the Gini-Simpson’s diversity of Iraqi FQREC was relatively higher than that of the US CIP-R (0.739 vs. 0.697), with a projected number of clonal groups of FQREC being 1.7 times higher in Iraq than in the USA (Supplementary Figure S1). The minor clonal groups comprised 22.9% of Iraq isolates but only 6.1% of USA isolates (*p* < 0.001 in the chi-square test). Interestingly, only three of the minor clonal groups were the same in Iraq and the USA, while six of the major clonal groups (comprising at least 3% in either Iraq, the USA, or both) were the same, though with some differences in relative prevalence, as follows.

In both countries, the most dominant was *E. coli H*30, comprising 49.5% vs. 43.3% in Iraq and the US, respectively (*p* = 0.267) (Figure 1; Supplementary Table S1). To date, the second largest clonal group among US strains was ST1193, with a considerable 33.4% prevalence that, in contrast to *E. coli H*30 and other FQREC strains, is characterized by a relatively low resistance level to 3GC (Supplementary Table S3). Surprisingly, no ST1193 isolates were found among the Iraq CIP-R isolates. In contrast, the second largest group among the latter was ST405 (10.1% isolates), which was significantly less prevalent among the US CIP-R isolates (2.7%; *p* = 0.002). Another group overrepresented among Iraq vs. USA CIP-R isolates was ST410 (4.6% vs. 0.3%, respectively; *p* = 0.002), while isolates belonging to ST131-non-*H*30 were more common in the USA than Iraq (3.8% vs. 0.0%, respectively). Other clonal groups of relatively major size—ST10, ST69, and ST648—did not differ in prevalence between the countries.

While all CIP-R isolates that were selected for our study were resistant to ciprofloxacin at 2 mg/L concentration, 19 isolates (17.4%) from Iraq, but only 2 (0.7%) from the US (*p* < 0.01) carried either no or an incomplete set of the three classical mutations in QRDG regions of gyrA and parC (Supplementary Table S2). Among the ‘nonclassical’ US strains (both from ST131-non-*H*30), one carried only one QRDR mutation (S83L in GyrA), and one carried two mutations (S83L in GyrA and S80R in ParC). Among such Iraqi isolates, 9 carried no QRDR mutations (four from ST10, one from ST69, and the rest from minor groups), and nine isolates carried a single mutation of a diverse nature in GyrA (3 from ST69 and the rest from minor groups) (Supplementary Table S2).

### 3.3 Antimicrobial resistance level of *Escherichia coli H*30

Iraq-derived *E. coli H*30 were overwhelmingly resistant to 3GC and TMP/STX, significantly more so than the US-derived *E. coli H*30 (see Table 2). In addition, more Iraq-and US-derived *E. coli H*30 were resistant to at least three antibiotics (100% vs. 78.7%), or 4 (85.2% vs. 60.6%), or 5 (66.7 vs. 30.7, *p* < 0.001 for all), with 3 of the Iraq (but none US) *H*30 resistant to IMI. At the same time, the resistance level of *E. coli H*30 in either country did not differ significantly from the resistance of non-*H*30 CIP-R strains, except for US-*E. coli H*30 3GC resistance higher than US non-*H*30 strains (Table 2).

**Table 2:** Antibiotic resistance of urinary *E. coli* from Iraq hospitals has increased significantly compared to *E. coli* from US hospitals for *H*30 and non-*H*30 CIP-R *E. coli*-carrying samples.

*E. coli* collection		*H*30-carrying samples			non*H*30 FQREC-carrying samples			*H*30 vs. non*H*30, *p* value^b^	
		Iraq	USA	*p* value^a^	Iraq	USA	*p* value^a^	Iraq	USA
No. (% All)		54 (37.8)	127 (13.5)	**<0.0001**	55 (38.5)	166 (17.7)	**<0.0001**	0.9031	**0.0131**
Resistance, %	3GC^c^	90.7	53.5	**<0.0001**	81.8	25.9	**<0.0001**	0.1761	**<0.0001**
	TS^c^	83.3	52.0	**0.0001**	76.4	59.6	**0.0253**	0.364	0.1893
	CIP^c^	100	100	n/a	100	100	n/a	n/a	n/a
	NIT^c^	16.7	9.4	0.1653	14.5	4.8	**0.0159**	0.76	0.1191
	IMI^c^	7.4	0	**0.0019**	7.3	0	**0.0005**	0.9789	n/a
	FOS^c^	9.3	5.5	0.3539	10.9	3	**0.0196**	0.7751	0.2852
	MDR-3^c^	100	78.7	**0.0002**	98.2	75.9	**0.0002**	0.3195	0.5667
	MDR-4^c^	85.2	60.6	**0.0012**	92.7	53.6	**<0.0001**	0.2085	0.2298
	MDR-5^c^	66.7	30.7	**<0.0001**	63.6	20.5	**<0.0001**	0.7399	**0.0449**

a Prevalence and resistance compared between Iraq and US urine samples using the chi-square test (*p* < 0.05 in bold).

b Prevalence and resistance were compared between CIP-S vs. CIP-R *E. coli* within either Iraqi or US samples using the chi-square test (*p* < 0.05 in bold).

c 3GC, 3rd generation cephalosporins, TMP/STX, trimethoprim/sulfamethoxazole, CIP, ciprofloxacin, NIT, nitrofurantoin, IMI, imipenem, FOS, fosfomycin; MDR-3, MDR-4 and MDR-5—multidrug-resistant to at least three, four, and five classes of antibiotics (including ampicillin/amoxiclav and tetracycline). The sample is considered resistant if at least one of the isolated *E. coli* is non-susceptible to an antibiotic.

**Figure 2:**
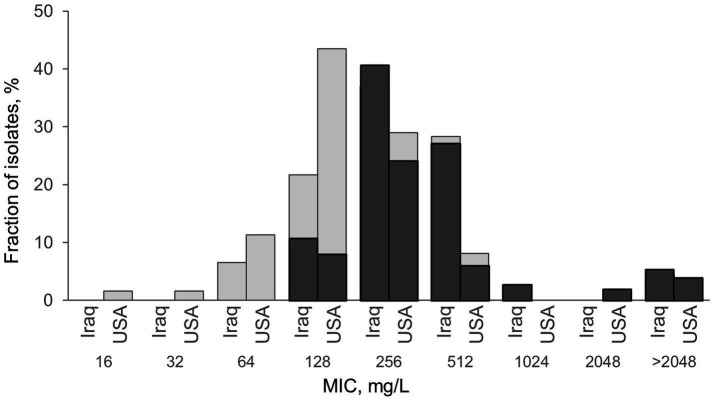
Comparison of *H*30 isolates from Iraq and the USA, with (dark gray) and without (light gray) PMQR, by the level of their resistance to ciprofloxacin. Minimum inhibitory concentration (MIC) was measured using the agar dilution method as described in Clinical and Laboratory Standards Institute (2022). For four isolates (2 from Iraq and two from the US) MIC was higher than the maximum 2048 mg/L and was assigned log_2_ = 12 for calculation purposes. PMQR loci *aac(6′)-Ib-cr* and *qepA* are described in Table 3. Both Iraqi isolates with MIC>2048 mg/L uniquely carry qepA. Exact *E. coli* counts and MIC values are listed in Supplementary Table S5.

Subsequently, we selected a random subset of *E. coli H*30 isolates from Iraq (*N* = 46) and the US (*N* = 62) (Figure 2) to determine the CIP minimum inhibitory concentrations (MICs). MICs ranged from 64 to >2048 mg/L in Iraqi *H*30 (log_2_[MIC] 6÷12) and from 16 to >2048 mg/L in the US *H*30 (log_2_[MIC] 4÷12). Four isolates (two from Iraq and two from the US) continued to grow at the highest tested CIP concentration, so they were assigned the next concentration value (refer to Methods). On average, Iraqi *H*30 tended to have higher MICs than US ones, with average log_2_[MIC] of 8.2 ± 0.2 and 7.5 ± 0.2, respectively, *p* = 0.010 (Figure 2). Since all of the *H*30 strains did not differ in the number or nature of QRDR mutations, we compared the strains for the presence of plasmid-mediated quinolone resistance (PMQR)-specific genes. Iraqi *H*30 isolates carried PMQR determinants twice as frequently as those from the US—69.5% versus 34.9%, respectively (*p* < 0.001) (Table 3), with the MICs of PMQR-negative strains significantly lower than for PMQR—positive strains in both countries—7.4 ± 0.3 vs. 8.5 ± 0.2, respectively, for Iraq (*p* = 0.003); and 7.0 ± 0.1 vs. 8.5 ± 0.3, respectively, for the US (*p* < 0.001) (Supplementary Table S5). When PMQR-negative and PMQR-positive subpopulations were separately analyzed, the difference between Iraqi and US *H*30 became nonsignificant. The predominant PMQR gene in *H*30 was almost exclusively *aac(6′)-Ib-cr*, with only two Iraqi isolates, with MICs above the maximum, having the *qepA* PMQR instead.

**Table 3:** Comparative analysis of *H*30 isolates from Iraq and US subclonal structure (Rx), antibiotic resistance, and presence of resistance determinants.

Characteristic^a^	Iraq	US	*p* value^b^
	*N* = 46 (%)	*N* = 63 (%)	
Rx clade	35 (76.1)	31 (49.2)	0.005
3GC-R	35 (76.1)	33 (52.4)	0.013
*bla_CTX-M15_*	35 (76.1)	23 (36.5)	<0.001
*bla_CTX-M27_*	7 (15.2)	10 (15.9)	0.926
*bla_TEM-1_*	8 (17.4)	29 (46)	0.003
*bla_OXA-1_*	31 (67.4)	22 (34.9)	0.001
TMP/STX-R	35 (76.1)	32 (50.8)	0.008
*dfrA1*	32 (69.6)	29 (46.8)	0.019
*sul1*	33 (71.7)	36 (58.1)	0.146
*sul2*	31 (67.4)	30 (48.4)	0.051
*dfrA1 & sul1*	33 (71.7)	37 (58.7)	0.164
*dfrA1 & sul2*	32 (69.6)	30 (47.6)	0.016
*qepA*	2 (4.3)	0	0.095
*aac(6′)-Ib-cr*	30 (65.2)	22 (34.9)	0.002

a Rx clade was determined by the presence of 264 and 260 SNPs (Price et al., 2013); 3GC-R and TS-R status were determined using the disk diffusion method (Clinical and Laboratory Standards Institute, 2022).

b *p* values were calculated in the chi-square test for 2×2 comparisons.

### 3.4 Genomic structure and content of *Escherichia coli H*30

To explore the evolutionary connections between *H*30 isolates from Iraq and the US, a subset of 46 Iraqi and 63 US *H*30 isolates underwent WGS. The CIP-R *E. coli H*30 strains from both countries were distributed between two clades—*H*30R (also known as C1) and *H*30Rx (also known as C2). *H*30Rx was more prevalent among Iraqi isolates than US isolates (76.1% vs. 49.2%; *p* = 0.005). Within each clade, Iraq isolates tended to form multiple minor sub-clades interspersed among the US isolates, suggesting endemic circulation without evidence for the emergence of a phylogenetically distinct major group of *H*30 isolates.

**Figure 3:**
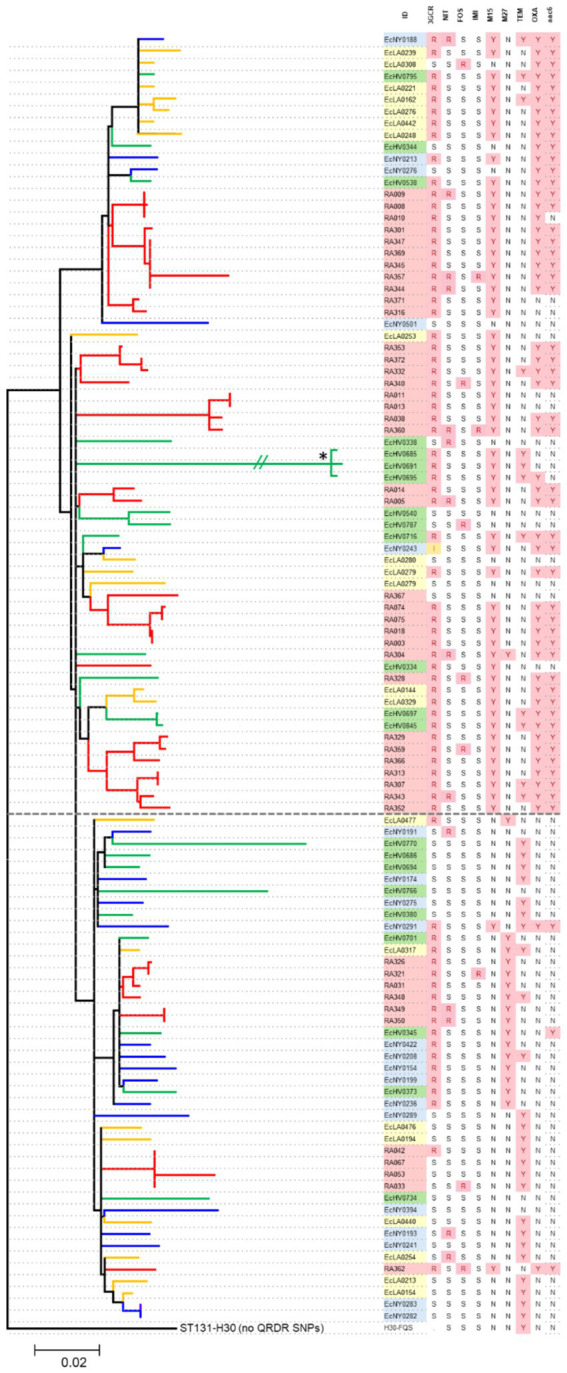
Phylogenetic analysis of *H*30 isolates from Iraq and US urine samples. Branches are colored to distinguish the source of an isolate between Iraq (red) or US-based hospitals in Seattle (green), Los Angeles (yellow), and New York (blue). The dotted line divides Rx (above) and R (below) clades. The table on the right lists isolates’ IDs, resistance to 3GC, *bla_CTX-M15_* (M15), *bla_CTX-M27_* (M27), *bla_TEM-1_* (TEM), *bla_OXA-1_* and *aac(6′)-Ib-cr* (aac6) ‘Y,’ yes, ‘N,’ no, ‘R,’ resistant, ‘S,’ sensitive, ‘I,’ intermediate, “data not available. Star ‘*’ indicates that the branch length was shortened from the original 0.1443 for visual purposes.

Indeed, whole genome analysis combined with multiple logistic regression revealed that the increased carriage of certain antimicrobial resistance determinants, such as the co-carriage of *aac(6′)-Ib-cr* and *bla_OXA-1_* genes, were more strongly associated with strains from the *H*30Rx clade than their country of origin (Figure 3). Moreover, resistance to 3GC was primarily determined by the presence of two ESBL genes—*bla_CTX-M-15_* (associated with *H*30Rx) and *bla_CTX-M-27_* (associated with *H*30R)— regardless of the country of origin.

In contrast, another ESBL gene, *bla_TEM-1_*, was detected in both Iraqi and US *H*30 strains without any clade-specific association. Similarly, resistance to TMP/STX, IMI, NIT, or FOS was not associated with either clade, with resistant isolates being phylogenetically dispersed throughout the tree. Moreover, no previously unknown genes were found to be responsible for any type of resistance, including TMP/STX or IMI resistance.

## 4 Discussion

This study investigated the antibiotic resistance pattern, clonality, and genetic determinants of the resistance among urinary *E. coli* isolated in two countries that differ in their geographical location, socio-economic backgrounds, and, most importantly, antibiotic usage practices. Iraq, situated in the Middle Eastern region, faces a situation where, despite having national guidelines for antimicrobials (Iraqi National Antimicrobial Guidelines, 2020), broad-spectrum antibiotics are frequently overprescribed by physicians and commonly dispensed by pharmacies without a prescription, resulting in widespread overuse and misuse of these medications (Alkadhimi et al., 2020; Kurmanji et al., 2021). In the USA, there are strict policies towards antibiotic use, with antimicrobials dispensed by prescription only and various antimicrobial stewardship programs implemented. We found that, as expected, the multidrug resistance is significantly higher in Iraq than in the US.

The Iraqi urinary *E. coli* isolates were found to be significantly more resistant towards CIP, 3GC, TMP/SXT, FOS, and IMI than the US isolates, correlated with a study of AMR load in 204 countries in 2019, with Iraq reporting 12,400 resistance-associated deaths (Murray et al., 2022). Alarmingly, 6% of Iraqi samples carried IMI-resistant *E. coli*, while IMI resistance was absent in US samples. IMI and other carbapenems are considered a “drug of last resort” for the treatment of infections caused by MDR pathogens, and the World Health Organization (WHO) listed carbapenem-resistant enterobacteria (CRE) as the highest-priority pathogen (Huang et al., 2024). Moreover, alarming is the more than 5-fold higher resistance level in Iraqi isolates to 3GC, another broad-spectrum antibiotic that, in the USA, is reserved for treating patients at high risk for severe infections and complications (Gupta et al., 2011). Our findings on the high 3GC resistance rate in Iraqi *E. coli* isolates align with a review that analyzed 100 studies from various Iraqi provinces, which also reported elevated resistance rates towards cefotaxime (76.5%) and ceftriaxone (75.9%) (Al-Dahmoshi et al., 2020). The high resistance rates to IMI and 3GC are likely due to extensive use of those antibiotics for prophylactic and empirical treatment in Iraq, with IMI prescribed in nearly 12% of all antibiotic treatment cases, while 3GC is the most prescribed class of antibiotics in general, comprising 37% of all prescriptions (Kurmanji et al., 2021).

In both countries, CIP-R strains were more often resistant to non-CIP antibiotics than CIP-S strains. The phenomenon that CIP-R strains tend to be multidrug-resistant is well known but not fully understood. Possibly, CIP-R *E. coli* could be more tolerant to the carriage of plasmids, the common vehicles of resistance genes (Rozwandowicz et al., 2018). Furthermore, CIP-R strains could be highly successful gut colonizers and, thus, tend to be exposed to many different antibiotics over a prolonged commensal carriage (Gurnee et al., 2015; Tchesnokova et al., 2023; Tchesnokova et al., 2020). Moreover, some QRDR mutations (in the gyrA S83 and parC S80 positions) were demonstrated to confer a fitness benefit onto the isolates that may permit the acquisition of an extra resistance gene cargo (Fuzi and Sokurenko, 2023).

Among CIP-R strains, the most dominant clonal group in both Iraq and the US was pandemic multidrug-resistant *E. coli H*30, comprising approximately half of the isolates in both countries. This is not surprising, considering the well-documented extensive spread of this clonal group in different regions of the world (Banerjee et al., 2013; Colpan et al., 2013; Johnson et al., 2013; Johnson et al., 2015b; Can et al., 2016a; Johnson et al., 2016; Can et al., 2016b; Kawamura et al., 2018; Miles-Jay et al., 2018), also possibly connected to its success as the gut colonizer (Gurnee et al., 2015). The *H*30Rx subclade of *E. coli H*30, which tends to carry ESBL gene *bla*_CTX-M-15_ and a cluster of another ESBL gene OXA-1 and PMQR determinant *aac(6′)-Ib-cr* in IS26 transposon, was more common among the Iraqi strains. However, this subclade is not specific to Iraqi *E. coli* as the other major subclade of *H*30—*H*30R that sometimes carries the ESBL gene *bla*_*CTX-M-27*_. Thus, the *E. coli H*30 strains comprise genetically well-known strain groups. Though no new major subclone appeared, the Iraqi *H*30 isolates tend to cluster in multiple small clades, suggesting an endemic mode of the strain circulation, i.e., that they are more or less geographically confined.

Surprisingly, the other major and globally spread CIP-R clonal group, ST1193, was absent from Iraqi samples comprising a third of US CIP-R strains, comparable size-wise to ST131-*H*30. ST1193 is a pandemic MDR clone known for its emergence in the late 1990s or early 2000s (Pitout et al., 2022). It was first reported to be isolated from patients in Australia in 2007–2008 and then spread worldwide. For example, ST1193 was reported among CIP-R strains isolated in the USA in 2011, China in 2011–2012, South Korea in 2013, etc. (Pitout et al., 2022). Moreover, it was reported from three countries neighboring Iraq (Neamati et al., 2020; Quan et al., 2021; Aljohani et al., 2023). ST131-*H*30 and ST1193 are the two most prevalent clones in unselected *E. coli* and the second most common clone in CIP-R *E. coli* (Tchesnokova et al., 2018; Tchesnokova et al., 2019b; Pitout et al., 2022). Hence, their complete absence from Iraqi samples in 2018–2019 is quite surprising. While it is possible that ST1193 had simply not yet “reached” Iraq by 2018, other barriers may have prevented its spread in the region. For example, because uropathogenic *E. coli*, including multidrug-resistant strains, derive from the gut resident microflora, the patients’ diet might affect the gut colonization prevalence of specific *E. coli* strains (Iacob et al., 2018; Leeming et al., 2019). Moreover, based on the US strains in our study set, ST1193 strains have a significantly lower level of resistance to 3GC relative to both *E. coli H*30 and other CIP-R strains that could impede the ST1193 spread in Iraq, where 3GC is the most broadly used antibiotic. Furthermore, the MIC values of ciprofloxacin in the Iraqi *E. coli* strains proved significantly higher than those in the American isolates. This observation is certainly linked to selection pressure caused by greater exposure to fluoroquinolones in Iraq and may also partly account for the lack of ST1193 strains in the Iraqi samples. The ST1193 strains of *E. coli* typically carry fewer QRDR mutations and have characteristically lower MIC values to fluoroquinolones (Pitout et al., 2022) than the ST131-*H*30 isolates harboring more QRDR mutations (Johnson et al., 2015a). Other demographic, socioeconomic, or other factors might also act as barriers to the spread of ST1193 in Iraq.

In contrast to ST1193, the second major CIP-R clonal group in Iraq, ST405, is nearly five times less prevalent than *E. coli H*30 but remains four times more common than ST405 in the US. ST405 is an emerging MDR clone and is a key contributor to the dissemination of extended-spectrum beta-lactamase (ESBL)-producing *E. coli* (Roy Chowdhury et al., 2018; Yao et al., 2022). The regional disparities in ST405 prevalence, as well as in the smaller ST131-non*H*30 (which is more prevalent in the US than in Iraq), are less pronounced compared to ST1193, though the reasons for these disparities remain unclear. Other global MDR clones in both Iraqi and US samples include ST10, ST69, ST648, and ST410, all of which are well-known international clonal groups. Overall, the clonal structure of CIP-R urinary *E. coli* in Iraq and the US shows no significant variation, with ST1193 being the only exception.

The QRDR mutations analysis shows that QRDR mutations, which are characteristic of the major ST131-*H*30 and ST1193 strains, were also carried by other clonal groups. Almost all minor ST and single ST isolates showing elevated MIC values to fluoroquinolones also carried gyrA S83 mutations, and many of them harbored additional parC S80 and GyrA D87 replacements. This proved typical for both US and Iraqi isolates.

Interestingly, it was demonstrated that less well-known QRDR mutations may confer higher resistance to *E. coli* than the most common gyrA S83 and parC S80 mutations (Lindgren et al., 2003). Moreover, it was reported that QRDR mutations different from these common alterations are more likely to evolve prior to them during the acquisition of resistance to fluoroquinolones (Cheng et al., 2020).

The question arises: why are the gyrA S83, D87, and parC S80 mutations much more common in fluoroquinolone-resistant isolates of *E. coli* than other QRDR alterations that may confer a higher resistance level and can evolve more rapidly?

The most probable explanation is that the gyrA S83 mutations confer a fitness benefit onto the isolates. All of the papers investigating the issue reported a fitness gain associated with the gyrA S83 mutations in both *E. coli* and *Salmonella* (Fuzi and Sokurenko, 2023). The parC S80 mutations were also observed to confer a fitness gain on many strains, but their impact is influenced by the genetic background of the isolates. Accordingly, the “double-serine” QRDR mutations (gyrA S83; parC S80) should confer an evolutionary advantage on multiple lineages of *E. coli* (Fuzi and Sokurenko, 2023).

Besides the QRDR mutations that provide high resistance to CIP, a relatively low resistance level can also be due to the overexpression of drug-specific efflux pumps or lower expression of outer membrane porins, which leads to decreased intracellular concentration of the drug (Alzrigat et al., 2017). Moreover, several plasmid-borne genes have been reported to mediate resistance, though again at a relatively low level. They include qnr genes (*qnrA*, *qnrB*, *qnrC*, *qnrD*, *qnrS*, and *qnrVC*) and those coding aminoglycoside acetyltransferase variants (*aac(6′)-Ib-cr*) and two efflux pumps (*qepA* and *oqxAB*) (Brandis et al., 2021). Qnr genes inhibit binding of FQs with DNA gyrase and topoisomerase IV; *aac(6′)-Ib-cr* variants reduce FQs efficacy by acetylating the drug, and QepA and OqxAB pump the drug out of the cell (Yamane et al., 2008). Thus, the higher MIC of Iraqi *H*30 could be explained by the higher frequency of *aac(6′)-Ib-cr*, which is the most prevalent plasmid-mediated FQ resistance mechanism that is known to provide clinical-level ciprofloxacin resistance in combination with other FQ resistance determinants (Gibson et al., 2010; Machuca et al., 2016). In this study, two *H*30 Iraqi isolates with a very high ciprofloxacin MIC (>2048 ug/ml) were found to harbor *QepA1*. QepA, first detected in clinical isolates of *E. coli* from Japan and Belgium, belongs to the major facilitator superfamily (MFS) of efflux pumps and has been reported to increase MIC to CIP by 32–64 fold (Perichon et al., 2007; Yamane et al., 2008). Since then, they have been reported worldwide (Cattoir et al., 2008; Rincón et al., 2014; Rahman et al., 2017). This finding confirms that although PMQRs by themselves may provide a relatively low-level resistance to quinolones, they help bacteria select chromosomal CIP resistance-determining mutations, which in turn can increase the MIC (Jacoby et al., 2014).

However, one limitation of the study is the lack of detailed information regarding the specific sources of the clinical urinary isolates. Nevertheless, we believe that the strain sets are fairly comparable because the clinical samples were provided randomly within consecutive days of submission from major centralized diagnostic laboratories serving large urban populations (both in Iraq and the US). All the samples were collected from patients suspected of UTIs, and both clinical and laboratory diagnoses were made according to similar standards in both countries.

## 5 Conclusion

Altogether, our research reveals a significant increase in antibiotic resistance among *E. coli* strains isolated from urine samples in Iraq compared to those in the US. This rise in resistance is evident in both CIR-R and CIP-S strains, with notably higher minimum inhibitory concentration (MIC) levels in Iraq due to the greater prevalence of plasmid-borne resistance genes. Additionally, while the diversity of CIP-R strain clones in Iraq surpasses that in the US, a considerable portion of strains in both countries belong to established international clonal groups or their subclades. This suggests that increased antibiotic usage promotes the spread of globally disseminated clonal lineages and well-known plasmid-mediated resistance genes rather than fostering the emergence of novel, highly successful strain groups. However, alongside these similarities, notable differences in the prevalence of specific *E. coli* clonal groups highlight the potential influence of geographic, demographic, cultural, dietary, or socioeconomic factors on the dissemination of specific bacterial strains.

## Data Availability

The datasets presented in this study can be found in online repositories. The names of the repository/repositories and accession number(s) can be found in the article/Supplementary material.

## Footnotes

^1^ https://enterobase.warwick.ac.uk/

^2^ https://www.bv-brc.org/

^3^ https://blast.ncbi.nlm.nih.gov/Blast.cgi
