# Supplementary figures and images for: Clonal and resistance profiles of fluoroquinolone-resistant uropathogenic Escherichia coli in countries with different practices of antibiotic prescription
Source: Front Microbiol. 2024 Oct 2;15:1446818. doi: 10.3389/fmicb.2024.1446818 (PMC11479919; doi:10.3389/fmicb.2024.1446818)

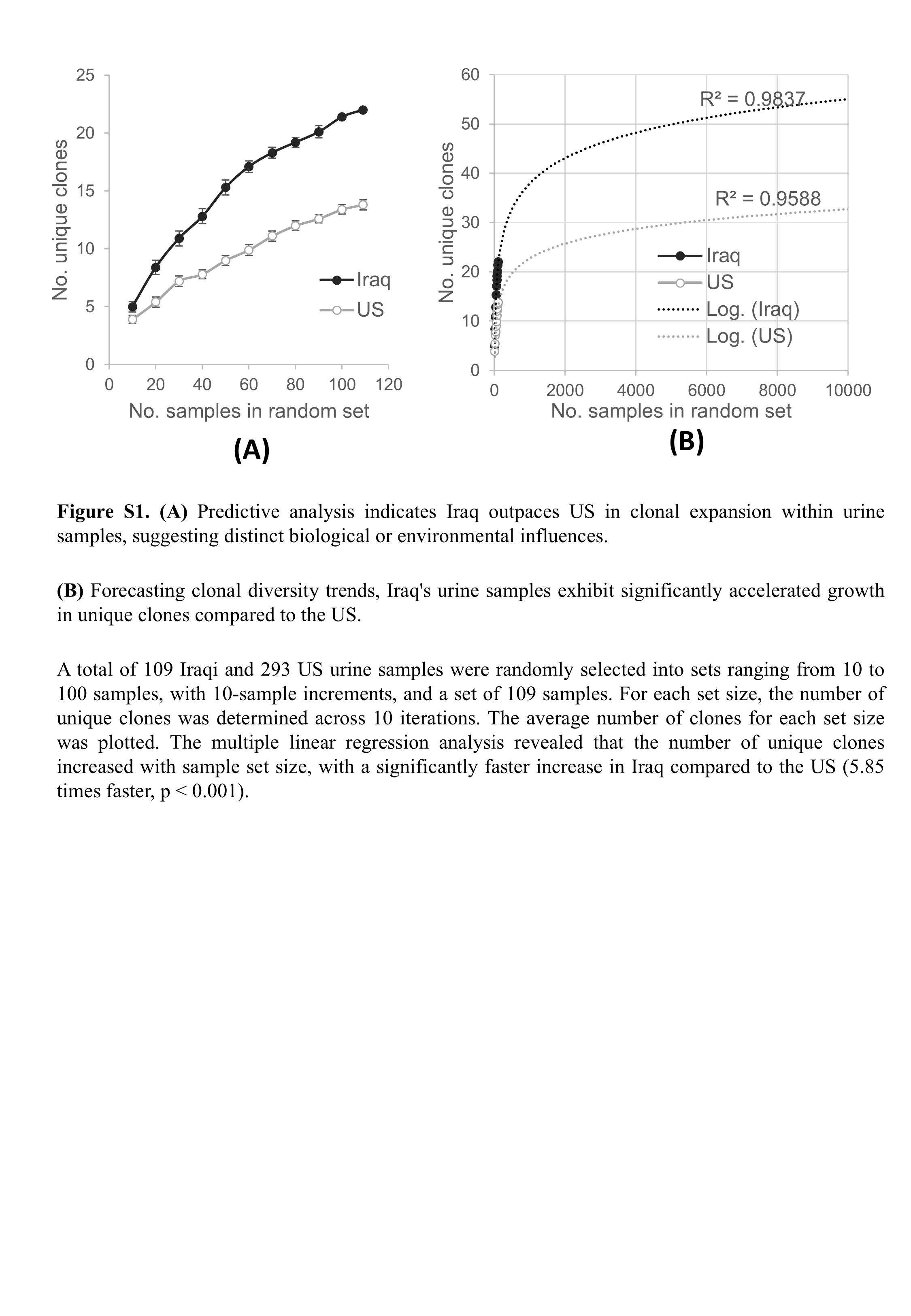

Supplement: Supplementary file 1 [file Image_1.tif]
