# Supplementary material for: Clonal and resistance profiles of fluoroquinolone-resistant uropathogenic Escherichia coli in countries with different practices of antibiotic prescription
Source: Front Microbiol. 2024 Oct 2;15:1446818. doi: 10.3389/fmicb.2024.1446818 (PMC11479919; doi:10.3389/fmicb.2024.1446818)
Supplement: Supplementary file 2 [file Table_1.DOCX]

Supplementary Material

**Table S1. Comparison of clonal structure of prevalent CIP-R *E. coli* isolated from urine samples from Iraq and US hospitals.** In bold – significantly higher prevalence (P < .05 in Chi-square test).

| **Clonal group** | **No. *samples* (% from N samples)** | |
| --- | --- | --- |
|  | **Iraq (N = 109)** | **USA (N = 293)** |
| ***H*30** | 54 (49.5) | 127 (43.3) |
| **ST1193** | 0 | **98 (33.4)** |
| **ST405** | **11 (10.1)** | 8 (2.7) |
| **ST10** | 7 (6.4) | 10 (3.4) |
| **ST69** | 4 (3.7) | 11 (3.8) |
| **ST648** | 3 (2.8) | 9 (3.1) |
| **ST131-nonH30** | 0 | **11 (3.8)** |
| **ST410** | **5 (4.6)** | 1 (0.3) |
| *‘Other’ minor clones* |  |  |
| **ST38** | 3 (2.8) | 2 (0.7) |
| **ST448** | **3 (2.8)** | 1 (0.3) |
| **ST73** | **3 (2.8)** | 0 |
| **ST156** | **3 (2.8)** | 0 |
| **ST1290** | **2 (1.8)** | 0 |
| **ST1196** | 1 (0.9) | 1 (0.3) |
| **ST354** | 0 | 2 (0.7) |
| **ST12** | 0 | 2 (0.7) |
| **ST4204** | 1 (0.9) | 0 |
| **ST224** | 1 (0.9) | 0 |
| **ST196** | 1 (0.9) | 0 |
| **ST141** | 1 (0.9) | 0 |
| **ST127** | 1 (0.9) | 0 |
| **ST457** | 1 (0.9) | 0 |
| **ST424** | 1 (0.9) | 0 |
| **ST16** | 1 (0.9) | 0 |
| **ST453** | 1 (0.9) | 0 |
| **ST345** | 1 (0.9) | 0 |
| **ST167** | 0 | 1 (0.3) |
| **ST361** | 0 | 1 (0.3) |
| **ST443** | 0 | 1 (0.3) |
| **ST349** | 0 | 1 (0.3) |
| **ST205** | 0 | 1 (0.3) |
| **ST95** | 0 | 1 (0.3) |
| **ST48** | 0 | 1 (0.3) |
| **ST372** | 0 | 1 (0.3) |
| **ST155** | 0 | 1 (0.3) |
| **ST393** | 0 | 1 (0.3) |
| **Total** | 109 | 293 |

**Table S2. Analysis of combinations of QRDR mutations in GyrA and ParC in Iraq and US CIP-R *E. coli*.**

| **Clone** | **GyrA** | **ParC** | **Iraq** ^a^ | **US** ^a^ |
| --- | --- | --- | --- | --- |
| ***H*30** | S83L, D87N | S80I, E84V | 54 | 63 |
| **ST1193** | S83L, D87N | S80I | 0 | 23 |
| **ST405** | S83L, D87N | S80I | 9 | 1 |
|  | S83L, D87N | S80R | 2 | 0 |
| **ST10** | none | none | 4 | 0 |
|  | S83L, D87N | S80I | 3 | 2 |
| **ST69** | none | none | 1 | 0 |
|  | S83A | none | 3 | 0 |
|  | S83L, D87N | S80I | 0 | 1 |
| **ST648** | S83L, D87N | S80I | 3 | 2 |
|  | S83L, D87N | S80I, E84G | 0 | 1 |
| **ST131-nonH30** | S83L | none | 0 | 1 |
|  | S83L | S80R | 0 | 1 |
|  | S83L, D87N | S80I | 0 | 2 |
| **ST410** | S83L, D87N | S80I | 5 | 1 |
| **Minor STs** | none | none | 4 | 0 |
|  | S83L | none | 5 | 0 |
|  | D87Y | none | 1 | 0 |
|  | S83L | S80I | 1 | 0 |
|  | S83L, D87N | S80I | 11 | 3 |
|  | S83L, D87Y | S80I | 1 | 0 |
|  | S83L, D87N | S80I, E84A | 1 | 0 |
|  | S83L, D87N | S80I, E84G | 0 | 1 |

^a^ All Iraq CIP-R *E. coli* and a random set of US CIP-R *E. coli* had *gyrA* and *parC* loci sequenced to determine presence and type of QRDR mutations.

**Table S3. Resistance of FQREC clones from Iraq and USA.**

| **Clone** | **Country** | **No.**  **(% CIP-R samples)** | **Resistance, %** | | | | | | |
| --- | --- | --- | --- | --- | --- | --- | --- | --- | --- |
|  |  |  | **CS3** | **TS** | **CIP** | **NIT** | **IMI** | **FOS** | **TET** |
| **H30** | Iraq | 54 (49.5) | **90.7** | **83.3** | 100 | 16.7 | 7.4 | 9.3 | 64.8 |
|  | USA | 127 (43.3) | 53.5 | 52 | 100 | 9.4 | 0 | 5.5 | 52.8 |
| **ST1193** | Iraq | 0 (0) | . | . | . | . | . | . | . |
|  | USA | 98 (33.4) | 12.2 | 56.1 | 100 | 4.1 | 0 | 1 | 50 |
| **ST405** | Iraq | 13 (11.9) | **92.3** | 84.6 | 100 | 7.7 | 0 | 7.7 | 92.3 |
|  | USA | 8 (2.7) | 62.5 | 75 | 100 | 0 | 0 | 25 | 87.5 |
| **ST10** | Iraq | 7 (6.4) | 85.7 | 42.9 | 100 | 42.9 | 14.3 | 14.3 | 71.4 |
|  | USA | 10 (3.4) | 20 | **60** | 100 | 0 | 0 | 0 | **70** |
| **ST69** | Iraq | 6 (5.5) | 16.7 | 33.3 | 100 | 0 | 0 | 0 | 16.7 |
|  | USA | 11 (3.8) | 72.7 | 81.8 | 100 | 0 | 0 | 0 | 72.7 |
| **ST648** | Iraq | 7 (6.4) | 57.1 | 71.4 | 100 | 0 | 0 | 14.3 | 57.1 |
|  | USA | 9 (3.1) | 55.6 | 44.4 | 100 | 0 | 0 | 11.1 | 100 |
| **ST131-nonH30** | Iraq | 1 (0.9) | 100 | 100 | 100 | 0 | 0 | 0 | 100 |
|  | USA | 11 (3.8) | 27.3 | 54.5 | 100 | 0 | 0 | 0 | 45.5 |
| **ST410** | Iraq | 5 (4.6) | 100 | 60 | 100 | 20 | 0 | 0 | 100 |
|  | USA | 1 (0.3) | 100 | 100 | 100 | 0 | 0 | 0 | 100 |
| **ST448** | Iraq | 5 (4.6) | 100 | 100 | 100 | 20 | 20 | 0 | 100 |
|  | USA | 1 (0.3) | 0 | 0 | 100 | 0 | 0 | 0 | 0 |
| **Other** | Iraq | 40 (36.7) | 57.5 | 65 | 97.5 | 5 | 5 | 7.5 | 55 |
|  | USA | 17 (5.8) | 41.2 | 70.6 | 100 | 23.5 | 0 | 5.9 | 41.2 |

**Table S4. Primers and probes used in the study.**

| Test | Target | Primer name | Sequence | Ref |
| --- | --- | --- | --- | --- |
| CH typing | **fumC: PCR1** | fumC-F | GCATCACAGGTCGCCAGCG | Tchesnokova et al., 2023 |
|  |  | fumC-R | GTACGCAGCGAAAAAGATTC |  |
|  | **fumC: Nested** | fumC-F'-T7Pro | TAATACGACTCACTATAGGGGCGCTTCAAATTTGTTCGG | Tchesnokova et al., 2023 |
|  |  | fumC-R'-T7Term | GCTAGTTATTGCTCAGCGGGTACGCAGCGAAAAAGATTC |  |
|  | **fimH: PCR1** | fimH-F | CTGTTTGCTGTACTGCTGATG | Tchesnokova et al., 2023 |
|  |  | fimH-R | CCACAATAAACGGTAAGAGGAAT |  |
|  | **fimH: Nested** | fimH-F'-T7Pro | TAATACGACTCACTATAGGGACTGCTGATGGGCTGGTC | Tchesnokova et al., 2023 |
|  |  | fimH-R'-T7Term | GCTAGTTATTGCTCAGCGGAGGAATTGGCACTGAACC |  |
| Detection of QRDR SNPs | **gyrA: PCR1** | gyrA-F | CGACCTTGCGAGAGAAAT | Tchesnokova et al., 2023 |
|  |  | gyrA-R | GTTCCATCAGCCCTTCAA |  |
|  | **gyrA: Nested** | gyrA-F'-T7Pro | TAATACGACTCACTATAGGGCGAGAGAAATTACACCG | Tchesnokova et al., 2023 |
|  |  | gyrA-R'-T7Term | GCTAGTTATTGCTCAGCGGAGCCCTTCAATGCT |  |
|  | **parC: PCR1** | parC-F | CGATTGCCGCCTGAGCCACTT | Tchesnokova et al., 2023 |
|  |  | parC-R | GCGAATAAGTTGAGGAATCAG |  |
|  | **parC: Nested** | parC-F'-T7Pro | TAATACGACTCACTATAGGGTGAGCCACTTCACGCA | Tchesnokova et al., 2023 |
|  |  | parC-R'-T7Term | GCTAGTTATTGCTCAGCGGGAGGAATCAGAATTAA |  |
| *H*30 specific probes | **Dual-labeled Probe, 5' HEX/3' BHQ-1** | H30-P4/6 [Hex] | CCCTTTGGCACCCAACCCAA | Tchesnokova et al., 2016 |
|  | **Forward primer for fimH30-reaction** | H30-F4 | AAGCTATCTCTGGAAGCGTCCCT | Tchesnokova et al., 2016 |
|  | **Reverse primer for fimH30-reaction** | H30-R4 | CCCAAGTGAATTTGAAGAAACGGAG | Tchesnokova et al., 2016 |
|  | **Dual-labeled Probe, 5' Quasar-705/3' BHQ-3** | P131_705 | AGCCAGTCGCGGAGTTGGATTAA | Tchesnokova et al., 2016 |
|  | **Forward primer for ST131-reaction** | 131F | CGTGACCTGTCCGTTCATAAGTAG | Tchesnokova et al., 2016 |
|  | **Reverse primer for ST131-reaction** | 131R | TGGCATCAAACACTCAAGAGAA | Tchesnokova et al., 2016 |
| *H*30Rx/R | **SNP-200** | Forward Primer | GACACCATGCGTTTTGCTTC | Price et al., 2013 |
|  |  | Reverse primer | TCGTACCGGCAACAATTGAC | Price et al., 2013 |
|  | **SNP-264** | Forward Primer | GTGGCGATTTCACGCTGTTA | Price et al., 2013 |
|  |  | Reverse primer | TATCCAGCACGTTCCAGGTG | Price et al., 2013 |

**Table S5. Comparison of Ciprofloxacin Minimum Inhibitory Concentration (MIC) of *H*30 *E. coli* isolates from Iraq and US.**

| **MIC** | | **Total, no.** | | **No PMQR, no.** | | **PMQR+, no.** | |
| --- | --- | --- | --- | --- | --- | --- | --- |
| **mg/L** | **log2** | **Iraq** | **US** | **Iraq** | **US** | **Iraq** | **US** |
| 16 | 4 | 0 | 1 | 0 | 1 | 0 | 0 |
| 32 | 5 | 0 | 1 | 0 | 1 | 0 | 0 |
| 64 | 6 | 3 | 7 | 3 | 7 | 0 | 0 |
| 128 | 7 | 10 | 27 | 6 | 23 | 4 | 4 |
| 256 | 8 | 17 | 18 | 2 | 6 | 15 | 12 |
| 512 | 9 | 13 | 5 | 3 | 2 | 10 | 3 |
| 1024 | 10 | 1 | 0 | 0 | 0 | 1 | 0 |
| 2048 | 11 | 0 | 1 | 0 | 0 | 0 | 1 |
| >2048 | 12 | 2 | 2 | 0 | 0 | 2 | 2 |
| **Total *H*30, no** | | 46 | 62* | 14 | 40 | 32 | 22 |
| **Ave MIC log2 ± SE** | | 8.2+/-0.2 | 7.5+/-0.2 | 7.4+/-.3 | 7.0+/-.1 | 8.5+/-.2 | 8.5+/-.3 |
| **P value Iraq vs US** | | .010 | | .183 | | .899 | |

** One US isolate EcNY0241 did not have its MIC measured, hence calculations for this table were done with 62 US isolates.*

**Figure S1.**

**(A) Predictive analysis indicates Iraq outpaces US in clonal expansion within urine samples, suggesting distinct biological or environmental influences.**

**(B) Forecasting clonal diversity trends, Iraq's urine samples exhibit significantly accelerated growth in unique clones compared to the US.**

**Data_S1: Core genes used to build the tree**

**Data_S2: wgMLST gene description**

**Data_S3: Isolates used in the study**

**Data_S4: Accessory genes different for same wgMLST strains**

**Data_S5: Uberstrain ID of whole genome sequenced isolates**
